# Lateral supramalleolar flap: Is it based on perforator of peroneal / anterior tibial artery; A cross-sectional study at tertiary care centre

**DOI:** 10.1016/j.amsu.2021.102916

**Published:** 2021-10-22

**Authors:** Pervaiz Mehmood Hashmi, Kamran Ahmed, Muhammad Ali, Abeer Musaddiq, Alizah Hashmi, Zohaib Nawaz

**Affiliations:** a Aga Khan, University hospital, Karachi, Pakistan; b Indus Hospital, Karachi, Pakistan

**Keywords:** Supramalleolar flap, Peroneal artery perforator, Foot, and ankle, Soft tissue defect

## Abstract

**Background:**

To determine the anatomical basis of supramalleolar flap; retrograde versus antegrade and its clinical outcome based on the vascular pattern.

**Methods:**

This analytic cross-sectional study was conducted at a tertiary care hospital in Karachi, Pakistan. Patients who underwent coverage of soft tissue defects around the foot and ankle with supramalleolar flaps were included. Data collection was through medical records including demographic parameters, mechanism of injury, per-operative findings of perforator origin, and patient interviewing for final assessment. Patients with peripheral vascular disease, unavailability of skin, and radiation injuries were excluded. All analysis was done using SPSS version 25.0.

**Results:**

49 patients were included in the study from May 1999 to December 2020. The male to female ratio was 37:12. The cause of soft tissue defects was trauma in 9 (38.7%) followed by Infection in 16 (32.6%) and Blast injury in 5 cases (10.2%). The maximum flap size harvested was 20 × 8 cm. In 19 cases the peroneal artery perforator was absent and the flap was based on the perforator of an anterolateral malleolar branch (antegrade) while the remaining 30 flaps were based on the perforator of the peroneal artery (retrograde**).** Overall, the flap survival rate was 98%; as 1 case had partial necrosis and required skin grafting. However, there were 9 minor complications. In 8 patients, the flap was rotated as a **‘*delay flap’***. All patients had satisfactory functional outcomes without significant morbidity of the donor site.

**Conclusion:**

The lateral supramalleolar flap provided coverage to almost all regions of the foot and ankle with a cosmetically acceptable donor and recipient site. There were no problems with shoe wear, as only 2 patients required defatting for cosmetic reasons. Microvascular expertise was required for a predictable outcome.

## Highlights

• This study describes anatomical basis of flap; antegrade vs retrograde. The antegrade flap was based on the inferolateral collateral branch of anterior lateral malleolar artery.
• The complications rate was higher in retrograde flaps based on the perforator of peroneal artery as compared to antegrade flap.
• Outcome of antegrade flap was better as it was much more vascular and robust.

## 1 Introduction

Soft tissue defects around the foot and ankle commonly occur as a result of road traffic accidents (RTA) that may involve bone, tendon, and neurovascular structures [1]. Other causes include blast injuries, machine/industrial injuries, resection of tumors, and neuropathic ulcers [2]. The incidence of such injuries in Karachi (an industrial and heavily populated city), is quite high because of a poor roads traffic system, the city ranked 4th in road traffic accidents in the world [3]. Road traffic accidents, gunshots, and bomb blast injuries frequently lead to open fractures with skin loss. Similarly, cases of chronic osteomyelitis, chronic burns, tumor excision, and skin necrosis due to irradiation around the foot and ankle require soft tissue coverage. Such cases present very late and are commonly associated with infections. These cases pose a great challenge due to underlying infections, limited local soft tissue availability, and the need for secondary reconstruction of involved structures (bone, tendon, nerves, and vessels). These complex wounds require staged reconstruction; initial skeletal stabilization followed by coverage with thin, pliable soft tissue, and subsequent repair/reconstruction of the tendon, bone, and neural tissue for restoration of limb functions [4,5].

Reconstruction of soft tissue defects of the foot and ankle depends on the location, size, and depth of the wound. Various local available options include local muscle flap, fascia-cutaneous flap, perforator flap, metatarsal artery flap, sural artery flap, and supra-malleolar flap [6,7].

Free skin grafts are often unsuitable because they tend to contract and have poor resistance to pressure. Local skin flaps would be preferable but are relatively small and have limited range and reliability [7]. Further compromise of a major vessel of the leg, like the anterior tibial or posterior tibial artery, is not always desired. Local muscle flaps like flexor digitorum brevis and abductor hallucis longus are often inadequate to reach problematic skin defect areas like the heel, tendon Achilles, and peri-malleolar region. Cross leg flaps require immobilization in cumbersome positions and free flaps have their own merits and demerits. The lateral supramalleolar flap described by Yoshimura and Masquelet [8,9] is a very good option to cover the defect around the dorsum of the foot, ankle, and peri-malleolar area. This flap is based on the last perforator of the peroneal artery that pierces the interosseous membrane just above the ankle syndesmosis and becomes continuous with the anterolateral malleolar artery. The cutaneous branch of this perforator supplies the skin over the anterolateral aspect of the lower one-third of the leg. Masquelet AC [9] in 1988 described the lateral supramalleolar fasciocutaneous flap based on a cadaveric and patient study. Without compromise to the major blood vessels of the foot, including the peroneal artery, this flap has proved to be a reliable fasciocutaneous flap for the locoregional coverage of the distal third of the leg, ankle, peri-malleolar region, dorsum, and plantar aspect of foot [[9], [10], [11]], except the weight-bearing region of the heel [10,12,13] as the flap is insensate and relatively thin. It can be a rather large flap (15 × 8 cm in clinical series [9] and 22 × 9 cm in dye injection studies in fresh cadavers [14]). The pedicle of the flap is long 8.0 cm in rotation flaps [9] to 15 cm in distally based flaps with compound pedicle [10] and the pivot point of the pedicle is sinus tarsi, which increases the arc of rotation. The flap is most commonly employed as a distally based pedicle island flap.

We did a retrospective analysis of supramalleolar flaps done for coverage of defects around the foot and ankle by a single surgeon over 21 years from May 1999 to December 2020. The main objective of our study was to evaluate the anatomical basis of lateral supramalleolar flap based on preoperative findings; is this flap is based on the perforator of the peroneal artery (retrograde) or based on ascending perforator (inferolateral malleolar artery) the anterolateral branch of the anterior tibial artery (antegrade). The second objective was to determine the clinical and functional outcomes of lateral supra-malleolar flap based on vascular pattern (retrograde versus antegrade) in terms of viability, coverage of defect, cosmetic appearance, and functions of the foot and ankle.

## 2 Patients and methods

This was an analytical cross-sectional study done by a single surgeon from 1999 to 2020, and included all the patients who required soft tissue coverage for defects around the foot and ankle. Data was collected through a structured proforma that included demographic parameters, causes of soft tissue defects, (secondary to open fractures, following debridement for chronic osteomyelitis, infected fractures, infected non-unions, tumor excision, pressure sores, and scar contracture as a result of old burns), site of defect, size, and type of flap (peninsular, antegrade, retrograde), per-operative findings, presence or absence of peroneal perforator and inferolateral collateral artery (anterolateral malleolar artery), complications and outcome of the flap. The clinical outcome data were gathered by patient interviews. The patients with advanced peripheral vascular disease, advanced diabetes mellitus, unavailability of skin, very large skin defects, radiation injuries, defects of the weight-bearing region of the heel, and defects reconstructed with other flaps were excluded from the study. All the patients were operated on with help of loupe magnification of 4.5 times. No preoperative Doppler or angiography was done to assess the state of vascular pattern of the flap. The protocol of the study was approved by the departmental research committee (DRC) and ethical review committee ERC. The final clinical and functional assessment was done by two research officers based on a self-assessment tool. This tool has four major parameters of assessment: percentage of coverage of defect by the flap, cosmetic appearance, weight-bearing status, and activities of daily life (ADL). The data were analyzed in SPSS version 25.0. The work has been reported in line with the STROCSS criteria [15].

## 3 Dissection technique

**Fig. 1:**
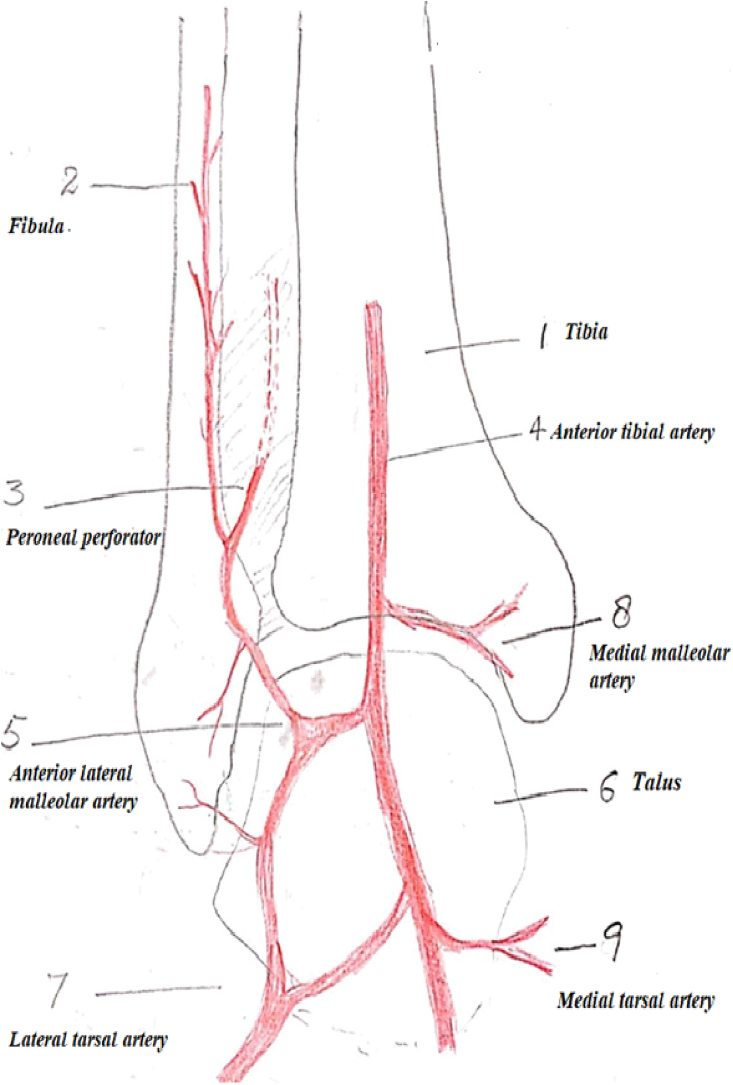
Showing schematic diagram of anastomosis of vessels around ankle and presence of Peroneal perforator. 1. Tibia 2. Fibula. 3. Peroneal perforator. 4. Anterior tibial artery. 5. Anterior lateral malleolar artery 6. Talus 7. Lateral tarsal artery. 8. Medial malleolar artery 9. Medial tarsal artery.

**Fig. 2:**
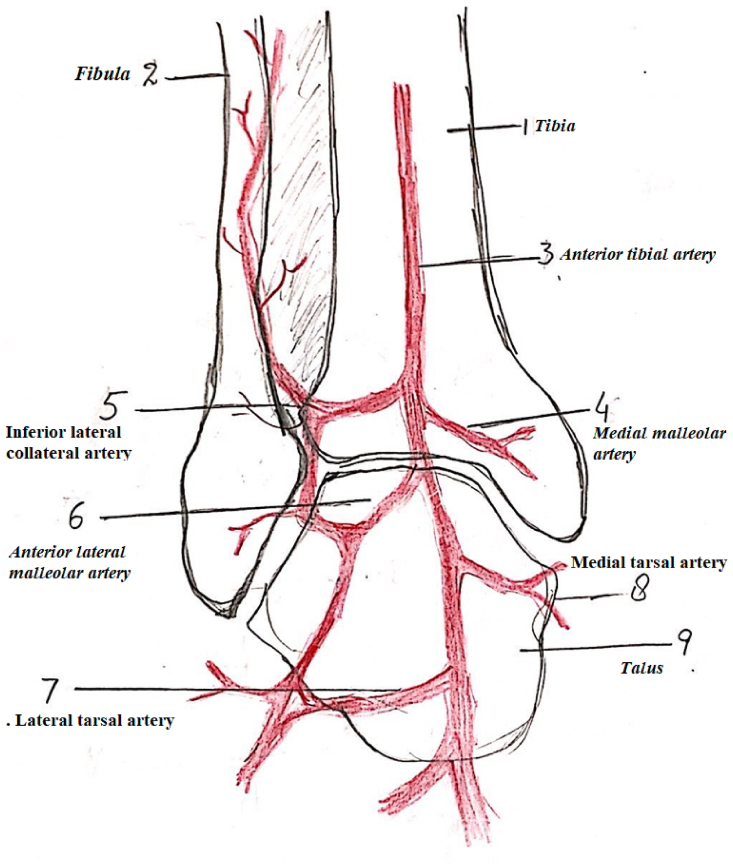
Showing schematic diagram of anastomosis of vessels around ankle and absence of Peroneal perforator and its replacement with inferolateral collateral artery. 1. Tibia 2. Fibula. 3. Anterior tibial artery 4. Medial malleolar artery 5. Inferior lateral collateral artery 6. Anterior lateral malleolar artery 7. Lateral tarsal artery. 8.Medial tarsal artery. 9.Talus.

**Fig. 3:**
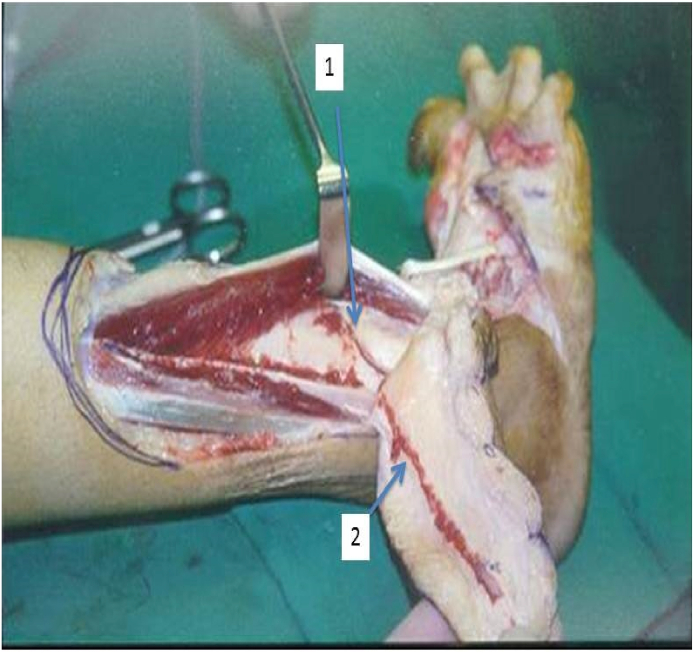
Showing Anterolateral malleolar artery (1) and its branch Inferolateral artery (2).

The patient is placed supine with a sandbag under the buttock and a pneumatic tourniquet on the thigh. The skin island is planned according to the defect. The distal limit should include the point of emergence of the perforating branch of the peroneal artery, four finger breadths above the lateral malleolus. The proximal limit reaches the mid-leg. The anterior limit is the tendon of the tibialis anterior muscle and the posterior limit should not cross the posterior border of the fibula. Then an incision is drawn anterior to the lateral malleolus and reaches the depression of the sinus tarsi on the lateral aspect of the hindfoot. First, the anterior margin of the flap is elevated, isolating the pedicle lying on the tibiofibular ligament. The superficial peroneal nerve is divided proximal to the flap and buried in the muscles. The pedicle is exposed. The posterior margin of the flap is then elevated. At this stage, the flap remains attached only to the septum between the anterior and lateral compartments. The perforating branch of the peroneal artery is then clamped temporarily to see the adequacy of retrograde flow in the skin island (Fig. 1). If the flow is good, then the perforator is ligated proximal to the emergence of skin perforators. If the flow is deemed unsatisfactory, the flap is sutured back on the bed and delayed for 48 h. In many cases, the peroneal perforator is absent and it is replaced by an ascending perforator called inferolateral collateral artery, a branch of the anterior lateral malleolar artery as shown in (Fig. 2). This ascending branch inferolateral collateral artery supplies the skin of the anterolateral aspect of the leg up to mid-level. Per-operative picture of such a case is shown in (Fig. 3) In the end, the septum is incised sub-periosteally and the flap rotated as a distally based pedicle island to the required defect. Release of the pedicle up to the sinus tarsi enhances rotation by adding length to the pedicle. Division of the fascia on the posterior border of the extensor digitorum brevis helps to avoid compression on the pedicle. The closure of the donor site is achieved by suturing the peroneal and extensor muscles together. A split-thickness skin graft is usually required for coverage of the donor site defect. In the case of peninsular rotation flap, the pedicle may not need exposure, and the flap rotated on a distal hinge. Similarly, for coverage of very distal defects on the bases of toes, a flap based on a compound pedicle can be harvested. In the case of an absent peroneal perforator, the flap may be based on antegrade circulation from the inferolateral collateral artery, a branch of the anterior tibial artery.

## 4 Result

A retrospective analysis of 49 patients who underwent reconstruction with soft tissue defects around foot and ankle with supramalleolar flaps was performed by the independent researcher over 21 years from 1999 to 2020. There were 37 (75.5%) males and 12 (24.5%) females with the mean age of 31.04 ± 20.6 years; ranging from 4 to 77 years The right to left side ratio was 27:22. Trauma was the major cause of soft tissue defect in 19 (38.7%) cases, followed by infections in 16 (32.6%), blast injuries in 5 (10.2%), tendon Achilles’ coverage in 4 (8%), contracture release in 3 (6%) and coverage of tumor defect in 2 (4%) cases. The various sites reconstructed with lateral supramalleolar flaps were: dorsum of foot in 26 (53%) cases, ankle in 12 (24.4%), and around the heel in 11 (22.3%) cases. (See Table .1). the mean follow-up of 13 years (ranging from 8 months to 20 years).

**Table 1:** Clinical summaries of 49 patients.

S.no	Variables		No. (%)
1	Gender	Male Female	37 (75.5%) 12 (24.5%)
2	Age	Mean ± SD (in years)	31.04 ± 20.6
3	Mechanism of injury	Trauma	19 (38.7%)
		Tumor	2 (4.0%)
		Blast injury	5 (10.2%)
		Infection	16 (32.6%)
		For Contracture release	3 (6.1%)
		For TA coverage s/p repair of TA	4 (8.1%)
5	Flap Size	Mean in cm (L + B)/2	9.15 ± 1.6
6	Complications	Flap Tip necrosis	3 (6.1%)
		Superficial epidermal marginal necrosis	5 (10%)
		Venous Congestion	5 (10%)
		Underlying Infections	2 (4%)
		No Complications	35 (71.6%)
7	Flap Survival		98%
8	Subsequent procedures (skin grafting for partial flap tip necrosis)		2%

Of these 49 cases, 35 (71.6%) cases showed complete healing with no complication. Flaps complications were recorded in only 14 (28.5%) cases of which 9 were minor complications. Significant venous congestion occurred in 5 cases and one had partial skin necrosis (20% of flap) requiring Skin grafting later on. Distal Superficial epidermal necrosis occurred in 5 cases which epithelized on its own. Flap tip necrosis was noted in 3 (6.1%) cases which also settled with dressings and epithelization without any procedure. In 8 cases the flap was retained on the donor bed after harvesting due to doubtful circulation on rotation to the recipient area. All these flaps were rotated electively as ‘DELAY FLAPS’ to the recipient area after 48 h ^(24).^

In 19 (38.7%) cases, the arterial blood supply was in antegrade circulation with inferolateral collateral artery, ascending perforator from an anterolateral malleolar artery, a branch of the anterior tibial artery due to the absence of the peroneal artery perforator. Only 3/19 (15.7%) of these cases showed complications like venous congestion (1), superficial epidermal marginal necrosis (1), and underlying infection (1) whereas no complications were seen in 16/19 (84.2%). However, 30 (61.2%) cases in which the Peroneal perforator was present and the arterial blood supply was in a retrograde fashion, the complications were observed in 11 (36.6%) cases i.e., 4 had venous congestion, 3 had tip necrosis, 4 had superficial epidermal necrosis and 1 had an underlying bone infection. One of three cases that had tip necrosis required skin grafting. All of these patients were followed up in the clinic till the time of complete wound healing and final ambulation. The average follow-up period was 162 ± 79.54 months (ranged from 8 months to 20 years, the average being 13 years).

Overall, the Flap survival rate was 98% based on complete flap coverage. The maximum flap size harvested was 20*8 cm which is larger than harvested by Masquelet AC (15 × 8 cm) in his clinical series [9]. The average operating time was 60–90 min and the donor site was covered with split-thickness skin graft in the same setting. There was no flap failure. Two patients required de-fattening of the flap for cosmetic reasons. The donor site was cosmetically acceptable to the majority of the patients.

For the long-term outcome analysis, the functional status was evaluated by a Self-Designed Tool in which outcomes of flaps in terms of coverage, cosmetic appearance, weight-bearing status, and activities of daily living (ADL) were assessed for each patient retrospectively and was given a certain score. The Excellent, Good, Fair, and Poor grades were given a score of 5, 4, 3, 2 (Refer to Table .2.). A collective score of 17–20 was graded as excellent, 13 to 16 as good, 9 to 12 as fair, and 8 as poor. Based on this clinical and functional evaluation, the majority of our cases fall in the “excellent” category – 41 (83.6%), good – 7 (14.2%), and fair – 1 (2%). Cosmetically, the texture and color of flaps were evaluated as good as compared with the original skin in 34 individuals, 10 patients reported it as “thick”, 2 complained of hairy skin and three patients labeled the skin as insensate. Regarding functions (ADL) and weight-bearing status, 44 (89.8%) patients were satisfied, 3 had problems in sports activities and shoe wear, and all had full weight-bearing status. The donor sites healed uneventfully and there were no poor cases in our study (Case 1, Case 2, Case 3, Case 4).

**Table 2:** Flap outcome grade by self-designed tool.

Variables	Excellent (5)	Good (4)	Fair (3)	Poor (2)
Coverage	100%	90–100%	80–90%	50–70%
Cosmetic Appearance	Highly acceptable	Acceptable with slightly raised skin margins	Acceptable with raised skin margins	Not acceptable due to thick and hairy skin
ADL	No issue in ADL and sports	No issue in ADL, difficulty in sports	The mild issue in ADL, cannot play sports	Difficulty in ADL and sports
Weight-bearing	Full weight-bearing	Full weight-bearing mild discomfort in sport	Discomfort in full weight-bearing	Pain on full weight-bearing
Total Score	20	16	12	8

(Showing Relation b/w flap coverages, cosmetic appearances, Daily life activities and Weight-bearing status of limb).

**Table 3:** Scorings based on Table 2 (Interpretation of Scores).

Outcomes Scores/Grades	No. of Patients (%) (N = 49)
Excellent	41 (83.6%)
Good	7 (14.2%)
Fair	1 (2%)
Poor	0
Mean Score ± S.D	19.0 ± 1.7

**Case 1:**
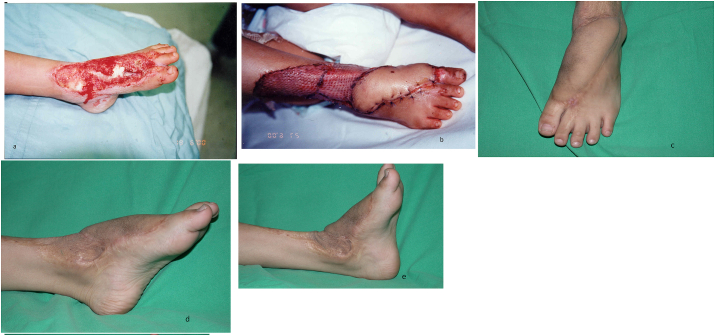
This 4 years girl sustained grade III-B fracture of first metatarsal of left foot as a result of runover injury. The area of degloving extended from dorsum of foot and ankle up to the bases of first and second toes (Figure 1.a). To cover this defect, lateral supramalleolar flap was raised as a retrograde pedicle island flap (Figure 1.b). Three and a half years postoperatively after de-fattening of the flap for cosmetic reasons (Figure 1.c), excellent range of ankle motion was achieved (Figure 1.d and 1.e).

**Case 2:**
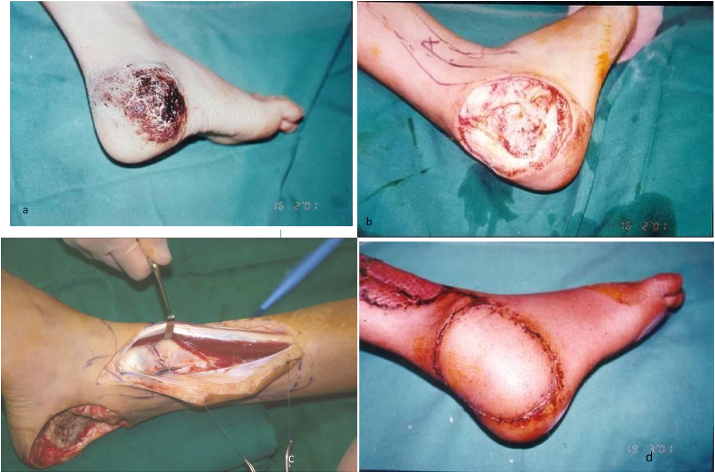
12 years male child, presented with a hemangioma on the lateral aspect of right heel for over two years with intermittent bloody discharge (Figure 2.a). Figure 2.b, showing defect. 2.c showing inferolateral collateral artery as the peroneal artery perforator was absent. 2.d showing the final outcome. Excellent functional and cosmetic result was achieved by excision of hemangioma and coverage with lateral supramalleolar flap.

**Case 3:**
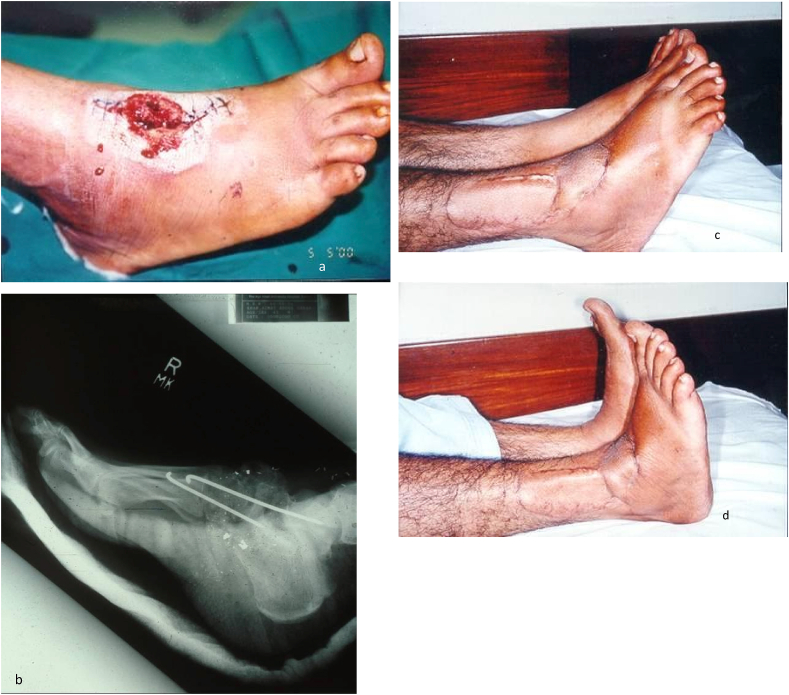
40 years old gentle man had gunshot injury to right foot with grade III open fracture of tarsometatarsal joints (Fig 3.a). Following debridement and skeletal stabilization, as shown in xrays (Fig 3.b). The defect was covered with Supramalleolar flap with good plantar and dorsiflexion (fig 3.c and 3.d).

**Case 4:**
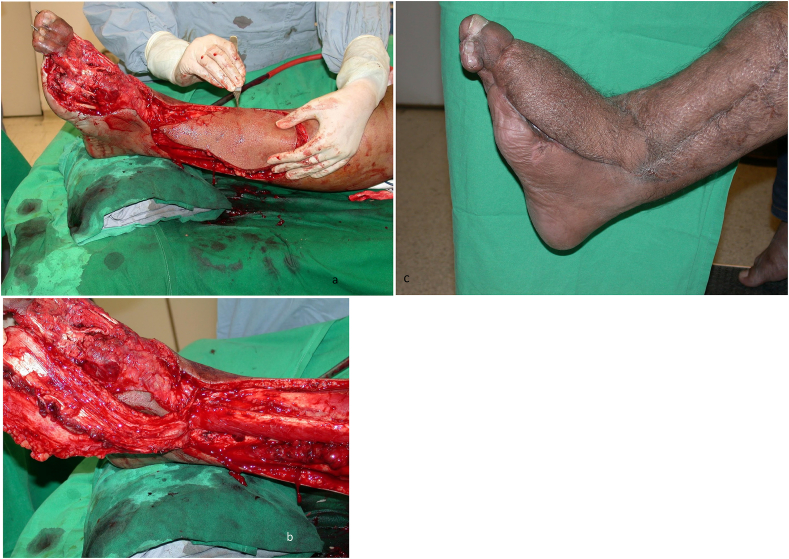
38 years old gentleman sustained injury to his left foot in road traffic accident when tyre of a vehicle ran over his foot. There was with loss of lateral two toes and skin over the dorsum of foot. Following debridement, defect was covered with supra malleolar flap, based in inferior lateral collateral artery (fig 4.a and 4.b). Excellent outcome after three years (fig 4.c).

## 5 Discussion

The potential problems of the flap [9] include some amount of venous congestion in reversed island flaps, painful neuroma as a result of a lesion of the superficial peroneal nerve, anatomic vascular variations [9,[14], [16], [17], [18], [19], [20], [21]], and the donor site concern, especially in young women. The usefulness of lateral supramalleolar flap has been demonstrated by its use as a delay flap [19,22] for tendon Achilles' coverage in difficult situations like Werner's syndrome, coverage of ischemic ulcer in Buerger's disease [20], as a free flap for oral coverage [14] and limb reconstruction [21].

Certain anatomic variations of the flap include the inconstant but frequent presence of a proximal inferolateral collateral artery [16,17], variations in the level of anastomosis between the perforating branch of the peroneal artery and the anterolateral malleolar artery arising from the anterior tibial artery, presence of the anterior peroneal artery, presence of a vascular network instead of a well-defined artery, absence of the perforating branch of the peroneal artery [16,17] and the basis of cutaneous circulation solely on anterior tibial perforators.

We encountered different variations in vascular anatomy as compared to other series. In 19 out of 49 cases the peroneal artery perforator was absent, (Fig. 2) which is a larger ratio and contradicts the previous data [9,16]. Dubreil Chambradel [8] found the peroneal artery perforator absent in 10 out of 165 cadaveric dissections. Relevant literature [9,[11], [12], [13], [14],[23], [24], [25], [26], [27]] shows a persistent and constant presence of perforator of the peroneal artery but our experience is different. We detected 19 cases out of 49 in which the peroneal artery perforator was replaced with inferolateral collateral perforator from anterolateral malleolar artery from the anterior tibial artery. This finding is consistent with the finding of Le Nen D et al. [16,17] who suggested that the inferior lateral collateral artery is the basis of lateral supramalleolar flap based on cadaveric and clinical studies. We think antegrade flap based on the inferolateral collateral artery is much more vascularized, robust, and perfused. The chances of its partial or tip necrosis are less, as we have seen in our study. In flaps based on the inferior collateral artery (a branch from the anterolateral malleolar), we noted that the artery bifurcated just like the perforator of the peroneal artery. The ascending branch of this inferolateral collateral artery supplies the anterolateral skin of the leg and descending branch anastomoses with the vessel of sinus tarsi, (Fig. 2) forming a continuous channel till the sinus tarsi. This arterial channel forms a basis for the flap.

We noticed that the incidence of complications was minimum in 19 cases of antegrade flaps where it was based on the antegrade circulation of inferolateral collateral artery from the anterior tibial artery. All complications were noted in those cases where peroneal artery perforator was present and the flap was based on the retrograde circulation and ascending branch of the peroneal artery perforator.

8 flaps needed to be left on the donor bed after harvesting due to doubtful circulation and later rotated to the recipient site as “DELAY FLAPS” after 48 h without any problems [22]. during this period the flap circulation improved due to opening of choke vessels at the periphery of flap. After the second procedure we did not notice any compromise in circulation, although 3 out of 8 had some congestion that settled over period of 4–5 weeks. None of these 8 flaps had skin necrosis after delayed rotation. Awareness of vascular variations is important and the use of delay flap in case of doubtful circulation is an excellent option. Even without Duplex imaging, the use of magnification was enough to evaluate the vascular anatomy per-operatively. It was possible to harvest the flap on all occasions despite vascular variations.

The lateral supramalleolar is not the first option for weight-bearing heel coverage [9,[11], [12], [13]], but the arc of rotation does allow one to cover the region in problem cases.

The majority of patients we encountered in our series were those with roadside accidents and bomb blast wounds, males being the major victims. Debridement followed by fracture stabilizations was done before flap coverage. Antibiotics were administered according to culture and sensitivities for an adequate period. Two patients required prolonged antibiotics therapy to control infection. Although many cases came to us after more than 12 hours of injury, we did not encounter any flap failure as a result of uncontrollable infection. We compared our results with the major case series of supramalleolar flaps done since its inception by Masquelet [2]. The comparison was done in terms of flap survival, complications, functional and aesthetic outcomes. The complication rate is variable in different series. Masquelet and Valenti P [9,11] have reported a complication rate of 7.6% each for venous congestion, partial necrosis, and hematoma formation, and a flap survival rate of 90%. C-Touam [23] reported a flap necrosis rate of 11% with a survival rate of 77%. The complication rate of flap necrosis and venous congestion was higher in older series [24,25] ranging from 5% to 20%. Recent series [26,27] show a relatively lesser rate of complications in terms of flap necrosis, venous congestion, and infections.

There are few pertinent points to our series worth mentioning. It is the largest case series by a single surgeon with a very long follow-up. The second aspect noted was that complications were associated with retrograde circulation (with perforator of peroneal artery) like partial or tip necrosis of flap; may be angio-some territory of ascending branch of the peroneal perforator is less/small as compared to ascending branch of the inferolateral collateral artery that supplies up to mid-leg (antegrade circulation). This aspect is well supported by other authors also [16,17]. The last feature of our case series is that we developed a self-designed tool to assess the clinical and functional outcomes of our flaps and patients. Four parameters were included cosmetic appearance, coverage of defect, the weight-bearing status of the foot, and ADL. Each variable was given four grades and four numbers correspondingly. The assessment was done by two independent researchers (doctors) who evaluated the patients in clinics and the final analysis of data was performed by an independent third party. The self-designed assessment tool and scoring system need validation by other researchers. This was an effort to determine the outcome of flaps objectively.

This flap can be raised as peninsular when a skin paddle is kept intact with a flap at the pivot point of rotation [28]. Most of the flaps are raised retrograde when the peroneal perforator is intact; the pedicle length can be increased along with its anastomosis with anterolateral malleolar artery and raised to sinus tarsi. We used this flap safely for the defects around the heel, tendon Achilles, peri-malleolar area, ankle joint, dorsum of foot up to toes, and plantar aspect of the foot except for the weight-bearing area of the heel. This is an excellent flap for coverage of the abovementioned defects provided the surgeon is familiar with anatomy, vascular variations, and microsurgical techniques. This procedure has to be done with patience as the dissection is tedious, just like the posterior interosseous artery flap in the upper extremity.

The understanding of fasciocutaneous flaps [21] and cutaneous vascular territories of the leg [29] has greatly aided in the development of flap surgery. Newer techniques and modifications of lateral supramalleolar flap as adipofascial flaps continue to emerge with good clinical success for small and medium-size defects [[30], [31]]. The study substantiates that the lateral supramalleolar fasciocutaneous flap is a reliable choice of flap for medium to large size wounds in the region of the peri-malleolar area, dorsum of foot, and ankle. There can be vascular variations [9,[14], [16], [17], [18]]. Although it is very old-fashioned flap but it is very useful in third world country where microsurgical skills are evolving and many budding surgeons are not familiar with free tissue transfer or Flap surgery. Therefore, color duplex imaging for preoperative localization of perforators and magnification for dissection around the vessel will greatly facilitate the outcome, especially for relatively less experienced microvascular surgeons. The usefulness of lateral supramalleolar flap for coverage of soft tissue defects around the foot and ankle is undoubted.

## Ethical approval

Approved by ERC committee and ERC number is 4452.

## Sources of funding

We have no affiliation with or financial involvement with any organization or entity with financial or other interest in the matter discussed in the manuscript.

## Author contribution

Dr Pervaiz Mehmood Hashmi: Study design and manuscript writing.

Dr Kamran Ahmed: Study design, data collection, and manuscript writing.

Abeer Musaddiq: study approval, data collection, study, manuscript writing, data analysis.

Dr Muhammad Ali: Data collection and manuscript writing.

Dr Zohaib Nawaz: Data collection.

Alizah Hashmi: contributors.

## Research registration number

Name of Registry: CHINESE Clinical Trial. Gov (ChiCTR).

Unique Identifying Umber or Registration ID: ChiCTR2100049970.

Hyperlink to your specific registration (must be publicly accessible and will be checked): http://www.chictr.org.cn/index.aspx.

## Guarantor

Dr Pervaiz Hashmi is the one who accepts the official responsibility of this research study that includes Ethics (approved by ERC), data handling, reporting of results and study conduct.

## Financial disclosure statement

The principal author has nothing to declare. No financial benefit or support was taken from any source.

## Data statement

Data will be made available on request.

## Provenance and peer review

Not commissioned, internally reviewed.

## Declaration of competing interest

None.
